# Mucosal Schwann cell hamartoma of the gallbladder

**DOI:** 10.4322/acr.2021.338

**Published:** 2021-10-22

**Authors:** Farhan Ismael, Sidrah Khawar, Ameer Hamza

**Affiliations:** 1 Dow University of Health Sciences Karachi, Department of Pathology, Karachi, Pakistan; 2 University of Kansas, University of Kansas Medical Center, Department of Pathology, Kansas City, Kansas, USA

**Keywords:** Schwann cells, Hamartoma, Neurofibroma, Neuroma, Gallbladder, MSCH

## Abstract

Mucosal Schwann cell hamartoma (MSCH) is a rare benign neurogenic tumor characterized by pure S100p positive spindle cell proliferation. Most cases occur in the distal colon. Involvement of the gall bladder is exceedingly rare. There have been no reports of recurrence or a syndromic association with MSCH. Herein, we describe a case of MSCH of the gallbladder in a 55-year-old female patient with prior history of gastrointestinal neurofibromas who presented with abdominal pain. MR imaging revealed choledocholithiasis, gallbladder thickening, and marked biliary and pancreatic ductal dilation. The patient subsequently underwent cholecystectomy with choledochoduodenostomy. Histologic evaluation of the gallbladder showed diffuse expansion of the mucosa with S100p positive cells with spindly nuclei and indistinct cytoplasmic borders and diagnosis of MSCH of the gallbladder was rendered.

## INTRODUCTION

Mucosal Schwann cell hamartomas (MSCH) are rare benign neurogenic mucosal based lesions composed purely of Schwann cells.^1^ They can arise sporadically anywhere in the gastrointestinal tract but are mostly found in the distal colon.^1^^,^^2^ They generally present as asymptomatic colonic polyps that are usually incidentally discovered on screening colonoscopy with no reported recurrences and syndromic association.^1^^,^^2^ Although MSCH is a well-established entity in the tubular gastrointestinal tract, they have been described in the gallbladder as well, albeit exceedingly rarely.^3^^,^^4^

Herein, we describe a case of gallbladder mucosal Schwann cell hamartoma in a 55-year-old female, with an emphasis on the pathological findings and differential diagnosis with neurofibroma, perineurioma, schwannoma, and ganglioneuroma.

## Case Report

A 55-year-old female with a past history of alcoholic cirrhosis, Wernicke's encephalopathy, tobacco abuse, and hypertension, presented to the emergency department for abdominal pain. Laboratory investigations revealed elevated total bilirubin (2.1 mg/dl; reference range[RR]: 0.3 to 1.2 mg/dl) and alkaline phosphatase (237 U/L; RR: 25 to 110 U/L). Abdominal ultrasonography revealed elongated nondistended gallbladder with mild wall thickening, trace pericholecystic fluid, and dependent layering sludge. No gallstones were identified. Mild intrahepatic and moderate extrahepatic biliary ductal dilatation with the common bile duct measuring up to 1.9 cm was noted. No obstructing choledocholith or mass was identified. Subsequently, multisequence and multiplanar MR imaging was obtained through the abdomen before and following the administration of gadolinium contrast material which showed, choledocholithiasis with associated marked biliary and mild pancreatic ductal dilatation, mild peribiliary hyperenhancement, and marked gallbladder wall distention with wall thickening. The hepatobiliary surgery team was consulted for surgical evaluation and management. The patient underwent cholecystectomy with choledochoduodenostomy. The gallbladder was sent for routine histologic evaluation.

**Figure 1:**
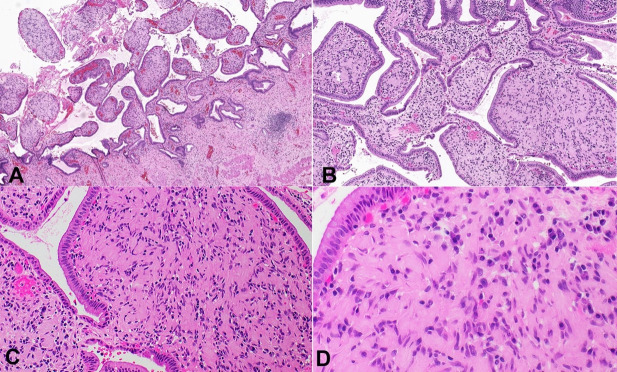
**A** – Low power photomicrograph demonstrating involvement of gall bladder mucosa (H&E 40X); **B-D** – High power photomicrographs showing cytologic details; cells with spindly nuclei and indistinct cytoplasmic margins (H&E 100X, 200X and 400X).

**Figure 2:**
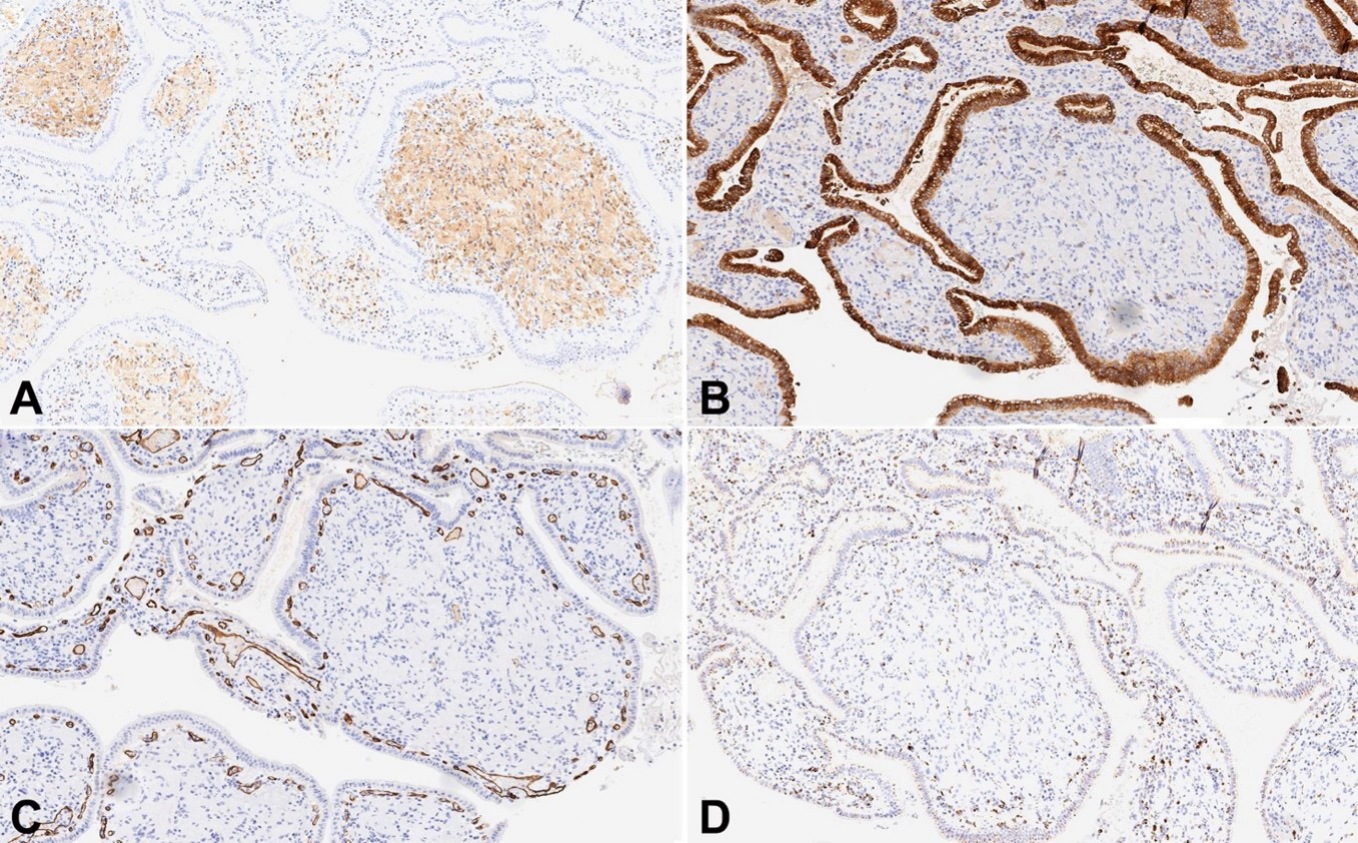
Immunohistochemical stains showing the lesional cells to be **A** – positive for S-100p; **B** – negative for EMA, **C** – CD34, and **D** – CD68.

Grossly the gallbladder was intact with a wall thickness ranging from 0.4-0.6 cm. The serosa was pink-tan smooth, with a roughened hepatic bed. Mucosa was tan-gray and hemorrhagic. No gallstones were identified. Histologic evaluation showed diffuse bulbous expansion of the mucosa with cells with spindly nuclei and indistinct cytoplasmic margins (Figures 1A-D).The proliferation was diffuse, unencapsulated, and diffusely replaced the lamina propria. Immunohistochemical stains showed that the lesional cells were positive for S100p while were negative for EMA, CD34, and CD68 (Figures 2A-D). Diagnosis of diffuse Schwann cell proliferation most compatible with Schwann cell hamartoma was rendered.

## LITERATURE REVIEW

We searched the PubMed and Scopus databases from their inception (1996 and 2004, respectively) to August 2021, using the keywords “Mucosal Schwann cell hamartoma of Gall Bladder”, “Gall bladder MSCH” and “Non-colonic MSCH of gastrointestinal tract”. This was followed by a manual search of the included references. Only two articles with confirmed involvement of gall bladder by MSCH were identified.

## DISCUSSION

Gibson et al.,^1^ first proposed the term Mucosal Schwann cell hamartoma (MSCH) for a group of gastrointestinal tract lesions that were purely composed of S100p positive spindle cells to differentiate them from true neuromas and neurofibromas. MSCHs are asymptomatic and are frequently identified as polyps on screening colonoscopy. They occur predominantly in the distal colon (rectosigmoid), with a few cases reportedly arising from the gastroesophageal junction and gastric antrum.^5^^,^^6^ These lesions are mostly found in middle-aged to elderly individuals and show a slight predilection for female patients.^1^^,^^7^^,^^8^ The MSCH-like lesion of the gallbladder has been recently described by Khanna et al.,^4^ predominately in young adults with a mean age of 25 years and by Sharma et al.,^3^ in a pediatric case. Our patient was 55 years old at the time of diagnosis.

Among the reported cases of gallbladder MSCH, no syndromic association or recurrence have been documented.^3^^,^^4^ Our patient had history of a gastrointestinal tract neurofibromas, and clinical suspicion of neurofibromatosis; however, confirmatory testing such as NF gene mutation analysis was not performed.

The exact etiology and precise pathogenesis of MSCH remain unclear. It is postulated that mechanical obstruction and chronic inflammation lead to the proliferation of the LGR5-positive intestinal stem cells and their plasticity may contribute to the hypertrophy of the nerve fibers in the gallbladder and gastrointestinal tract, eventually developing into an MSCH.^9^

The histological differential diagnosis for MSCH includes neurofibroma, schwannoma, perineurioma, and ganglioneuroma. Neurofibromas are usually associated with neurofibromatosis and present as a mural mass.^10^ They contain heterogeneous cellular components including Schwann cells, perineurial cells, fibroblasts, and axons, and rarely involve the mucosa.^1^^,^^10^ The mixed composition is evident on immunohistochemistry whereby they show varying degrees of expression of S100p, NFP and EMA.^10^ MSCH by contrast consists only of Schwann cells and shows diffuse S100p positivity, as found in our case.

Both schwannomas and Schwann cell hamartomas are composed of a pure population of Schwann cells, however, schwannomas usually show circumscription, perilesional lymphoid aggregates, collagenous stroma and nuclear heterogeneity whereas MSCH lack these features.^10^

Perineuriomas are benign nerve sheath tumors composed of perineurial cells. Like MSCH, mucosal perineuriomas are usually identified as polyps on routine or screening colonoscopy and they arise frequently in the left colon, particularly in the rectosigmoid.^1^^-^10 Perineuriomas show whorls of bland spindle cells with slender nuclei and elongated cytoplasmic processes, frequently entrapping colonic crypts. They are positive for EMA and Claudin-1 and are negative for S100p on immunohistochemistry.^1^^,^^10^

Ganglioneuromas are composed of a mixed population of S100p-positive Schwann cells, NFP–positive axons, and ganglion cells. The latter two components are absent in the mucosal Schwann cell hamartomas.^10^

Table 1 summarizes the morphologic and immunohistochemical differences between MSCH and its differential diagnoses.

**Table 1:** Differential diagnosis of mucosal Schwann cell hamartoma

**Lesion**	**MSCH**	**Neurofibroma**	**Schwannoma**	**Perineurioma**	**Ganglioneuroma**
**Common GI tract location**	Rectosigmoid colon	Rare in GI tract	Stomach followed by colorectum	Rectosigmoid colon	Oral cavity and tongue. Rare in GI tract proper
**Syndromic association**	None	NF1	None	None	MEN2B, NF1, Cowden
**Morphology**	Plump spindle Cells without elongated cytoplasmic processes.	Mixed population of Schwann cell, perineurial cells, fibroblasts, and axons.	Circumscribed spindle cell lesion with peripheral lymphoid cuff and collagenous stroma.	Whorls of bland spindle cells with slender nuclei and elongated cytoplasmic processes.	Mixed population of Schwann cells, axons, and ganglion cells.
**IHC**	S100p + EMA -	Variable positivity for S100p, NFP and EMA	S100p + Variable GFAP expression. Scattered CD34 + cells	EMA and Claudin-1 + S100p -	S100p-positive Schwann cells, NFP–positive axons

IHC = Immunohistochemistry; MSCH = Mucosal Schwann cell hamartoma, NF1 = Neurofibromatosis 1

Mucosal Schwann cell hamartomas of the gallbladder are extremely rare. There are no confirmed reports of gallbladder MSCH in patients with neurofibromatosis. Although MSCH does not have a syndromic association, the natural history of this lesion in patients with neurofibromas or clinically suspected neurofibromatosis is not yet elucidated.
